# Effect of Silica Based Nanoparticles against *Plasmodium* *falciparum* and *Leishmania infantum parasites*

**DOI:** 10.3390/jox11040011

**Published:** 2021-11-16

**Authors:** Ioannis Tsamesidis, Evgenia Lymperaki, Chinedu O. Egwu, Georgia K. Pouroutzidou, Konstantina Kazeli, Karine Reybier, Sandra Bourgeade-Delmas, Alexis Valentin, Eleana Kontonasaki

**Affiliations:** 1 Pharmadev, UMR 152, Université de Toulouse, IRD, UPS, 31000 Toulouse, France; echojay2010@yahoo.com (C.O.E.); karine.reybier-vuattoux@univ-tlse3.fr (K.R.); sandra.bourgeade-delmas@ird.fr (S.B.-D.); alexis.valentin@univ-tlse3.fr (A.V.); 2 Department of Biomedical Sciences, International Hellenic University, 57022 Sindos, Greece; evlimper@gmail.com (E.L.); kazeli.konstantina@gmail.com (K.K.); 3 School of Dentistry, Faculty of Health Sciences, Aristotle University of Thessaloniki, 54124 Thessaloniki, Greece; gpourout@physics.auth.gr (G.K.P.); kont@dent.auth.gr (E.K.); 4 Department of Biochemistry, College of Medicine, Alex-Ekwueme Federal University Ndufu-Alike Ikwo, Abakaliki PMB 1010, Nigeria; 5 School of Physics, Faculty of Sciences, Aristotle University of Thessaloniki, 54124 Thessaloniki, Greece

**Keywords:** silica-based nanoparticles, anti-parasitic properties, malaria, leishmanisis, artemisinin

## Abstract

Malaria and Leishmaniasis are two major parasitic diseases, endemic in large areas of tropical countries with high morbidity and mortality across the world. Nanoparticles in small sizes are specifically considered in medicine due to their ability to enter the cells, control the distribution of the administered drug and carry the drug specifically to the place of action. The present study aims to introduce the application of silica nanoparticles as new promising nanotools in malaria and leishmaniasis treatment. Ion doped silica nanomaterials revealed antileishmanial activities indicating the positive role of calcium, magnesium and copper to the surface of the particles against *Leishmania* parasites. Artemisinin-loaded nanoparticles presented the most promising antiparasitic properties with a sustained release able to overcome the parasite invasion. The sustainable release of artemisinin guarantee both the maintenance of its potential efficacy and also introduce an administration of drug to avoid subsequent drug resistance.

## 1. Introduction

Malaria and leishmaniasis are two important diseases of the tropics caused by *Plasmodium* and *Leishmania*. Malaria and leishmaniasis are responsible for at least 400,000 and 20,000 deaths annually, respectively [1,2,3]. The prevalence of these diseases has stalled and called for renewed strategy in the control approaches. Sometimes, these diseases co-exist as has been reported [4]. Several interventions have been in use; however, these are threatened by the development of resistance to the gold standard drugs for these diseases [3,5]. Resistance to these drugs is multifactorial: short half-lives, high cost that prevents complete treatment or drug overuse. Circumventing these shortcomings can offer hope for the control of these diseases. It is therefore pertinent to design and develop newer malarial and leishmaniasis drugs that can steady the fight against these tropical diseases. Nanotechnology has promises that can overcome some of these challenges [6]. Infusing the active ingredients into carriers that can intermittently release the drugs can overcome the short half-life and ensure full anti-parasitic effect of the drugs [7]. This work is therefore aimed at developing and testing the efficacy and safety of newer antimalarial and anti-leishmaniasis drugs using the nanotechnology. Nanocarriers application in drug delivery is breakeven research and has received a clarion call in biomedicine globally. Moreover addition of magnesium and copper in nanoparticles (NPs) appeared to improve the hemocompatibility [8,9] and in the literature it is not still demonstrated the effect of ions to counteract malaria or leishmaniasis. World health organization recommends artemisinin (ART) based combination therapies for the cure of malaria [3], and moreover ART appears in the literature to be active against *Leishmania* [10]. Artemisinin delivery *via* nanocarriers could prevent the development of resistance. The aim of our study is firstly to evaluate the antiparasitic properties of undoped and doped NPs and secondly to load nanocarriers with artemisinin and evaluate their synergistic effect with the ions doped to their surfaces in order to reduce the parasite functions.

## 2. Materials and Methods

### 2.1. Synthesis of Silica Based Nanoparticles (SbNs)

Silica-based nanoparticles, SiO_2_ (100% mol) (SbN1), SiO_2_CaOMgO (55, 35 and 10% mol, respectively) (SbN2), SiO_2_CaOMgOCuO (60, 30, 7.5 and 2.5% mol, respectively) (SbN3) were successfully synthesized by the Stöber-based sol–gel method. All samples were produced by the hydrolysis of TEOS in a mixture of double-distilled H_2_O, ethanol and HNO_3_, as previously described by our group [11,12]. Ca, Mg and Cu source were added as nitrate salts, while ammonia solution was added drop by drop under stirring and ultrasonic bath. All samples were dried at 75 °C for 2 days and calcinated in different temperatures (Table 1).

The synthesis of silica-based mesoporous nanocarriers (100% mol SiO_2_) (mSbN), was performed through a modified sol–gel method, as described before [13]. The cetyltrimethylammonium bromide (CTAB) was used as the agent for the mesoporous structure. CTAB was added in a solution of sodium hydroxide (NaOH, alkaline medium), and stirred for 30 min. Then tetraethyl orthosilicate (TEOS) was added dropwise and stirred vigorously for 2 h. The synthesized sample was dried at 60 °C overnight and calcinated at 600 °C for 5 h to remove CTAB.

### 2.2. Fourier Transform Infrared Spectroscopy (FTIR)

Perkin Elmer Spectrometer Spectrum 1000 was used for the Fourier Transform Infrared Spectroscopy (FTIR) analysis. The spectra of the synthesized samples were collected in the transmittance mode in the range of 400–4000 cm^−1^. The pellets of all samples with KBr powder (ratio of 1:100 ) were prepared under pressure of 7 tons.

### 2.3. ART-Loaded NP (ART-mSbN) and UHPLC/HRMS Analysis of ART Concentration

Mesoporous NPs in alkaline solution was used for the loading of 1 mM artemisinin after stirring (300 rpm) at room temperature for 1 day. The ART-mSbN were separated from the suspension by centrifugation at 5000× *g* for 15 min and was analyzed onto a UHPLC Kinetex EVO C18 1.7 µm, 2.1 × 100 mm column (Phenomenex, France) using UHPLC/HRMS system as previously described [14]. The following equation was used to calculate the loading capacity (LC) of artemisinin in NPs.

$$\mathrm{Loading}\;\mathrm{capacity}=\frac{\mathrm{total}\;\mathrm{amount}\;\mathrm{of}\;\mathrm{drug}\times \mathrm{free}\;\mathrm{amount}\;\mathrm{of}\;\mathrm{drug}}{\mathrm{weight}\;\mathrm{of}\;\mathrm{nanoparticules}}$$

For the drug release studies, supernatants at different time points (0, 6, 17, 21, 24, 41, 48, 72, 96 h) were collected and stored into the deep freeze. The artemisinin amount was quantified as previously described. The samples of each experiment were analyzed in triplicate.

### 2.4. Blood Sample Collection

Whole blood from healthy donors was collected after obtaining the informed consent to participate in the study. The separation of the erythrocytes from plasma and leukocytes was performed by washing them three times with Phosphate buffer saline (PBS). The study was conducted in accordance with Good Clinical Practice guidelines and the Declaration of Helsinki and was approved by Etablissement Français du Sang (EFS, Toulouse, France), responsible for ethic statements.

### 2.5. Antileishmanial Activity on L. infantum Axenic Amastigotes

*L. infantum* promastigotes (MHOM/MA/67/ITMAP-263, CNR *Leishmania*, Montpellier, France, expressing luciferase activity) were cultivated in RPMI 1640 medium supplemented with 10% fetal calf serum (FCS), 2 mM L-glutamine and antibiotics (100 U/mL penicillin and 100 μg/mL streptomycin) and harvested in logarithmic phase of growth by centrifugation at 900× *g* for 10 min. The acidified promastigotes were incubated for 24 h at 37 °C in a ventilated flask to transform promastigotes into amastigotes. The effects of the tested nanoparticles on the growth of *L. infantum* amastigotes were assessed as follows: 

*L. infantum* amastigotes were incubated at a density of 2 × 10^6^ parasites/mL in sterile 96-well plates with various concentrations of compounds dissolved in PBS (final concentration less than 0.5% *v*/*v*), in duplicate. Appropriate controls, PBS and amphotericin, were added to each set of experiments. After a 48 h incubation period at 37 °C, each plate-well was then microscopically-examined to detect any precipitate formation. To estimate the luciferase activity of axenic amastigotes, 80 μL of each well were transferred to white 96-well plates, Steady Glow^®^ reagent (Promega, London, UK) was added according to manufacturer’s instructions, and plates were incubated for 2 min. The luminescence was measured in Microbeta Luminescence Counter (PerkinElmer, Rome, Italy).

### 2.6. Antiplasmodial Activity

For the in vitro testing of the efficacy of the nanoparticles, the *Plasmodium falciparum* FCB1 strain was used. The parasites were kept in RPMI 1640 at 5% serum at 2% hematocrit at 37 °C 5% CO_2_ atmosphere. For each experiment, the parasites were tightly synchronized by 5% D-sorbitol treatment at the ring stage (0–24 h) (Lambros and Vanderberg, 1979). The inhibitory concentration (IC_50_) of the molecules were determined by light microscopy method as the gold standard method by WHO [15,16]. Tightly synchronized rings at 1% parasitemia were treated with different concentrations of each nanoparticle preparation, using artemisinin as control molecule and incubated for 48 h. The molecules were then washed off three times in PBS.

### 2.7. Assessment of Parasitemia by Light Microscopy

The evaluation of the parasitemia and the parasite stage was performed with thin smears and prepared at specific time points (24 h of incubation) and stained with Diff-Quick stain. Three observers examined a minimum of 10,000 cells microscopically. The experiments were performed at least in triplicate.

### 2.8. IC_50_ Measurement

Antiplasmodial activity: The program, ICEstimator software version 1.2, was used to estimate the half maximal inhibitory concentrations of the different nanoparticles estimates using a nonlinear regression function of the R software version 1.2.

Antileishmanial activity: Efficient concentration 50% (EC50) was defined as the concentration of drug required to inhibit by 50% the metabolic activity of *L. infantum* amastigotes compared to control. EC50 values were calculated by non-linear regression analysis processed on dose response curves, using TableCurve 2D V5 software. EC50 values represent the mean of three independent experiments.

## 3. Results and Discussion

### 3.1. Physicochemical Characteristics of SbNs

**Figure 1:**
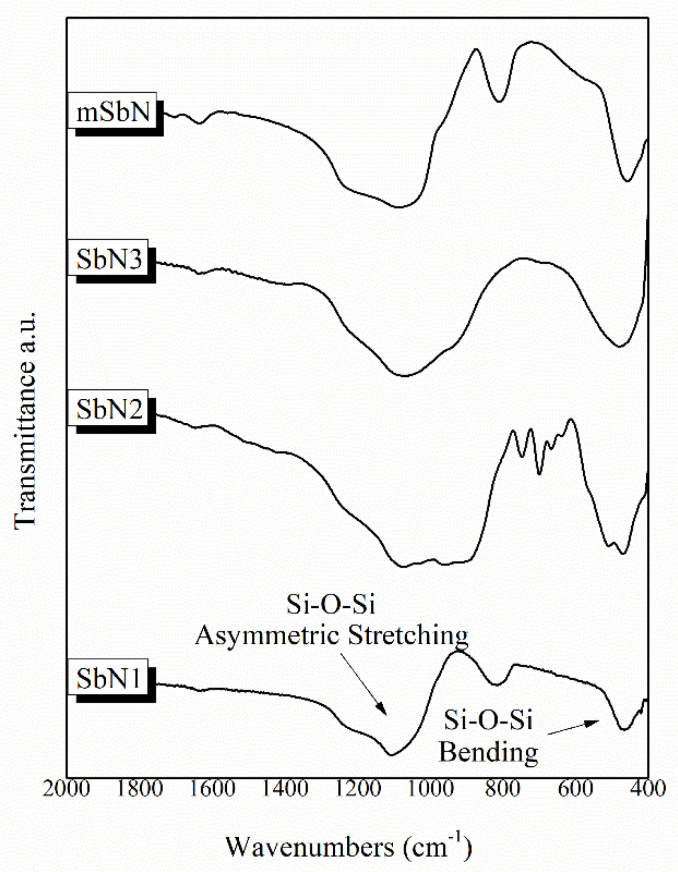
FTIR spectra of the synthesized samples.

The synthesized nanoparticles were of round shape with sizes of 2.05 nm ± 0.39 for SbN1, 6.82 ± 1.27 for SbN2 and 5.70 ± 1.06 for SbN3 and 408.80 ± 0.05 for mSbN [8,9]. All samples presented negative ζ-potential with the highest value recorded for the sample mSbN [8,9]. The FTIR spectra of the synthesized nanoparticles revealed the characteristic bands of silicate glasses in all spectra, assigned to the broad peak around 900–1200 cm^−1^ attributed to the symmetric stretching vibration of Si–O–Si and the peak around 470 cm^−1^ attributed to the bending vibration of the Si–O–Si bonds (Figure 1) [11,12,13]. Silica nanoparticles (SbN1) and mesoporous nanoparticles (mSbN) presented an additional peak around 814 cm^−1^ attributed to the symmetric stretching of the Si–O–Si bonds. Moreover, the spectra of SbN2 and SbN3 presented a shoulder around 902 cm^−1^ related to the vibration of the Si–O–Ca bonds [13].

### 3.2. Anti-Leishmania Activity of Nanoparticles

**Figure 2:**
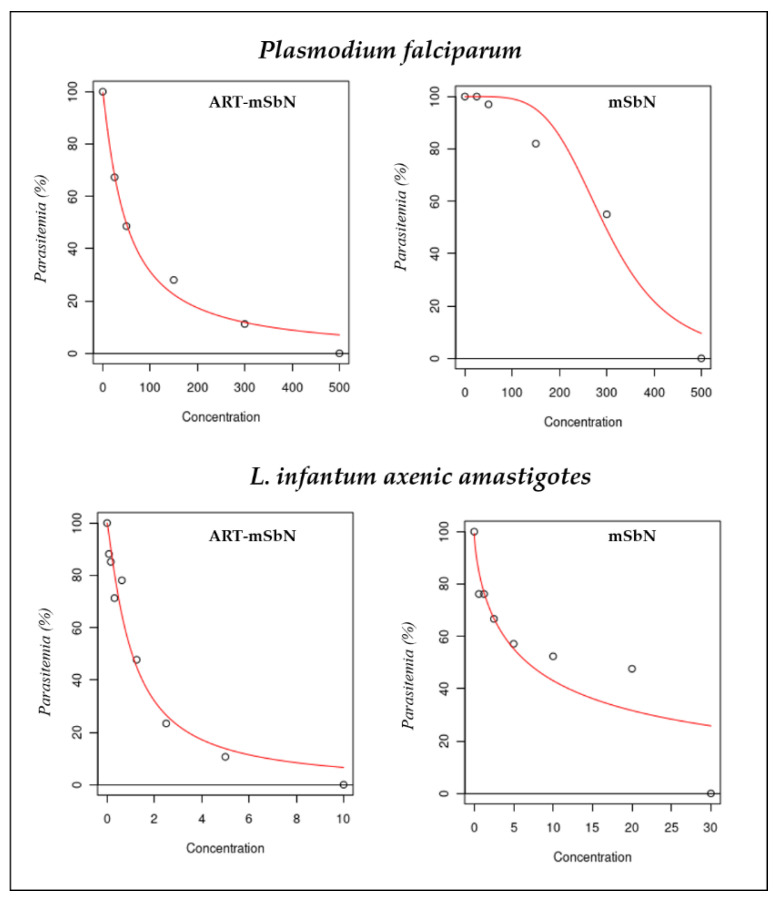
Rapresentation of the half maximal inhibitory concentrations (IC50s) non-linear regression curve of ART-mSbN and mSbN for *Plasmodium falciparum* and *L. infantum* axenic amastigotes.

The anti-leishmanial activity of the tested nanoparticles are presented in Table 2 using amphotericin B as positive control. The IC_50s_ were generally low in comparison to the gold standard, amphotericin B. The most effective was ART-mSbN, with an IC_50_ of 1.43 µg/mL which was lower than unloaded *mSbN* (10 µg/mL) (Figure 2). The anti-leishmanial effect of artemisinin has already been demonstrated [10], but was firstly presented *via* nanocarriers to counteract the parasites. This shows the nanotechnology absorbed the activity of ART to reduce the *L. infantum* promastigotes activity. However, a reported resistance to some antileishmanial drugs including artemisinin [17,18] calls for a paradigm shift in the administration and choice of drugs. An improved delivery through nanotechology could be fully exploited in the development of newer agents in the treatment of *Leishmania*. From all the nanoparticles, the SbN3 presented IC_50_ values close to the values of ART-mSbN. This particular nanoparticle was copper-doped. This activity maybe correlated to copper ions, as copper has been used as a chemotherapeutic agent against various parasitic infections [19]. In the study of Singh et al., a copper compound (copper salisylaldoxime–CuSAL) presented potent anti-leishmanial activity [20], while in a recent study, growth media supplemented with copper resulted in a gradual decline in *Leishmania* parasites survivability with increasing copper concentration [21]. More research is needed on investigating possible routes for utilizing copper or copper-doped nanoparticles towards chemotherapy of human leishmaniasis.

### 3.3. Antimalarial Activity of Nanoparticles

**Figure 3:**
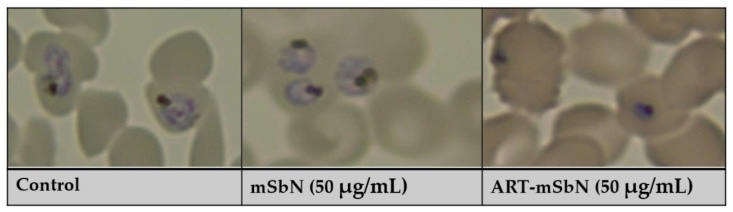
Morphological changes in *P. falciparum* after treatment with mSbN and ART-mSbN.

The antimalarial activity of the tested nanoparticles was evaluated after 24 h of incubation and is presented in Table 3 using artemisinin as positive control. As expected, of all the nanoparticles tested, *ART-mSbN* was the most effective (IC_50_ = 50 µg/mL) while the least effective was SbN1. The IC_50_ of the NPs are generally less than 500 µg/mL. The highest efficacy recorded from *ART-mSbN* could be indicative of a retention of the artemisinin activity that was embedded in the mesoporous structure of these nanoparticles (Figure 2). The morphology of parasites was significantly affected and is presented in Figure 3. ART-mSbN treated parasites showed detectable effects to their maturation and ability of invasion. The release capacity of the *ART-mSbN* was recorded and presented a sustained release until the first 48 h (Table 4). A slow-going release at the first 6 h started followed by a continuous release, eventually reaching a 69% as artemisinin release capacity. 

Artemisinin and its derivatives remain the gold standard in the treatment of malaria [3]. However its short halflife challenges its future usefulness in malaria control, as *Plasmodium* easily develop resistance due to short period of exposure. A technology that can intermittently release the molecule, overcoming the shortlife shortfall will be useful in the fight against resistance development to artemisinin. Nanotechnology has been reputed for this intermitent releases and targetted drug delivery of molecules [22], ensuring a sustained activity of different substances. These preliminary results suggest that by tailoring the properties of mesoporous nanoparticles, a prolonged and sustained release can be achieved, promoting the beneficial action of artemisinin. Future research should focus on mechanisms for increased grafting of artemesinin into and onto the surface of mesoporous nanoparticles for efficient drug loading and release. Taking into consideration, the involvment of intra-erythrocytic superoxide and hydrogen peroxide generation to activate artemisinin [23], the administration of artemisinin could be better enhanced *via* this system.

It is important to note that the above pharmacological activities of the tested nanoparticles was achieved without them having hemolytic effects as previously presented by Tsamesidis et al. [8,9]. The same set of hemocompatibility experiments was performed to confirm the biocompatibility of NPs in parastizied erythrocytes too (Supplemental Figure S1). All the tested biomaterials presented a biocompatible behaviour (hemolysis < 2%) which is considered as non-hemolytic according to ASTM F 756-08 (ASTMF-756, 2009). This underscores the safety of these silica-based nanoparticles as potential agents in the tretament of lesihmania and malaria, although further research is needed on whole blood components, such as platelets and white blood cells. 

## Figures and Tables

**Table 1:** Silica-based nanobioceramics (SbNs).

Silica Based Nanoparticles (SbNs)		
Nanoparticles (NPs)	Composition (mol %)	Calcination Temperature (°C)
SbN1	100% SiO_2_	700
SbN2	55-35-10% SiO_2_CaOMgO	1000
SbN3	60, 30, 7.5 and 2.5% SiO_2_-CaO-MgO-CuO	700
mSbN	100% SiO_2_	600

**Table 2:** Effects of nanoparticles against *Leishmania* amastigotes *in vitro*.

Nanoparticles/Drug	IC_50_ (µg/mL)
Amphotericin B	6.5 × 10^−2^
SbN1	>10 ± 1.1
SbN2	>10 ± 1.52
SbN3	2.46 ± 0.35
mSbN	>10 ± 0.91
ART-mSbN	1.43 ± 0.25

**Table 3:** Antimalarial effect of nanoparticles.

Nanoparticles/Drug	IC_50_ (µg/mL)
Artemisinin	9.8 nM = 2.8 × 10^−3^
SbN1	325 ± 35
SbN2	200 ± 28
SbN3	225 ± 15
mSbN	300 ± 29
ART-mSbN	50 ± 15

**Table 4:** Artemisinin release capacity (%) of ART-mSbN in a time range of up to 96 h.

Time (hours)	Artemisinin Release Capacity (%) of ART-mSbN
6	25
17	40
24	42
41	54
48	62
72	67
96	69

## Data Availability

Data are contained within the article.

## Supplementary Materials

The following are available online at https://www.mdpi.com/article/10.3390/jox11040011/s1, Figure S1: Hemolytic effect of NPs in parasitized erythrocytes.
