# Supplementary figures and images for: Effect of Silica Based Nanoparticles against Plasmodium falciparum and Leishmania infantum parasites
Source: J Xenobiot. 2021 Nov 16;11(4):155–62. doi: 10.3390/jox11040011 (PMC8628922; doi:10.3390/jox11040011)

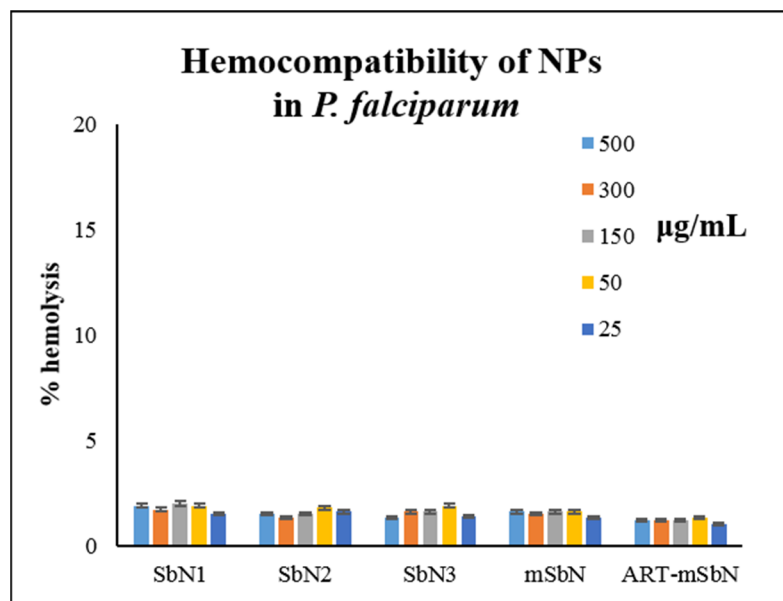

**Figure S1.** Hemolytic effect of NPs in parasitized erythrocytes.

Supplement: Supplementary file 1 [file jox-11-00011-s001.zip › jox-1424270-supplementary.pdf]
